# Intracellular Cleavage of the Cx43 C-Terminal Domain by Matrix-Metalloproteases: A Novel Contributor to Inflammation?

**DOI:** 10.1155/2015/257471

**Published:** 2015-09-03

**Authors:** Marijke De Bock, Nan Wang, Elke Decrock, Geert Bultynck, Luc Leybaert

**Affiliations:** ^1^Physiology Group, Department of Basic Medical Sciences, Ghent University, 9000 Ghent, Belgium; ^2^Laboratory of Molecular and Cellular Signaling, Department of Cellular and Molecular Medicine, KU Leuven, 3000 Leuven, Belgium

## Abstract

The coordination of tissue function is mediated by gap junctions (GJs) that enable direct cell-cell transfer of metabolic and electric signals. GJs are formed by connexin (Cx) proteins of which Cx43 is most widespread in the human body. Beyond its role in direct intercellular communication, Cx43 also forms nonjunctional hemichannels (HCs) in the plasma membrane that mediate the release of paracrine signaling molecules in the extracellular environment. Both HC and GJ channel function are regulated by protein-protein interactions and posttranslational modifications that predominantly take place in the C-terminal domain of Cx43. Matrix metalloproteases (MMPs) are a major group of zinc-dependent proteases, known to regulate not only extracellular matrix remodeling, but also processing of intracellular proteins. Together with Cx43 channels, both GJs and HCs, MMPs contribute to acute inflammation and a small number of studies reports on an MMP-Cx43 link. Here, we build further on these reports and present a novel hypothesis that describes proteolytic cleavage of the Cx43 C-terminal domain by MMPs and explores possibilities of how such cleavage events may affect Cx43 channel function. Finally, we set out how aberrant channel function resulting from cleavage can contribute to the acute inflammatory response during tissue injury.

## 1. General Aspects of Matrix-Metalloproteases and Their Role in Inflammation

Metzincin matrix-metalloproteases (MMPs) comprise a large family of endopeptidases of which today, 24 distinct genes have been identified in man (only 23 have been identified in mouse) [1]. The prefix “metallo-” refers to the reliance of these endopeptidases on zinc ions to perform hydrolysis of their respective protein substrates. MMPs are best known for their actions in remodeling of extracellular matrix (ECM) proteins and typical classification of the MMPs is based on their ECM substrate, their primary structure, and their subcellular localization. Later, MMPs were named according to their historic order of discovery. Groups of MMPs thus include the collagenases (MMP-1, MMP-8, and MMP-13), stromelysins (MMP-3 and MMP-10), stromelysin-like MMPs (MMP-11 and MMP-12), matrilysins (MMP-7 and MMP-26), membrane-type MMPs (MT-MMP-1 to MT-MMP-6), GPI-type MMPs (MMP-17 and MMP-25), and, probably the best known, gelatinases (MMP-2 and MMP-9) [2–4]. Research dedicated to identifying MMP targets has however uncovered that in fact the prefix “matrix” is far from complete as the MMP substrate repertoire is much more diverse and also includes growth factors, hormones, cytokines, and chemokines. Even more, MMPs are now known to also cleave intracellular targets (see later) [2, 3]. This multitude of target proteins grants MMPs involvement in a wide array of cellular functions. As such, they contribute to cellular differentiation and migration, regulation of growth factor activity, and cell survival as well as apoptosis, angiogenesis, and inflammation [1].

MMP activity is controlled at three levels: (i) transcription; (ii) proenzyme activation, and (iii) inhibition by endogenous proteins, most notably the “tissue inhibitors of metalloproteases” (TIMPs; TIMP-1 to TIMP-4). MMPs are synthesized as zymogens that are activated while being located intracellularly (see further), bound to the plasma membrane, or after secretion in the extracellular environment, most commonly by removal of their propeptide domain. A cysteine residue in the propeptide domain interacts with the catalytic zinc ion, thereby preventing protease activity until the propeptide domain is removed. The dissociation of this cysteine-Zn^2+^ interaction (“cysteine switch”) is a critical step in the activation of all MMPs. The third and fourth MMP protein domains are a linker region of variable length and a hemopexin domain that confers substrate specificity [2, 5]. TIMPs bind to the catalytic subunit of MMPs and inhibit them with a 1 : 1 stoichiometry (note though that there are just four TIMPs for over twenty MMPs) and the protease-antiprotease paradigm states that the net MMP proteolytic activity is the difference of total active MMPs minus the total TIMP activity [6]. Internalization, protease activity, posttranslational modifications (S-nitrosylation, glycosylation, oxidation, and alkylation), compartmentalization, and availability of substrates add additional levels of MMP activity control [1, 3].

MMPs are implicated in many physiological as well as pathological conditions, but we here focus on their role in inflammation. The inflammatory response is characterized by a cascade of molecular events including the secretion of cytokines, chemokines, and proteases by the damaged tissue as well as by infiltrating mast cells and neutrophils which are the sentinels responsible for detecting tissue damage or infection. This acute response subsequently promotes invasion of leukocytes from the blood side into the inflamed tissue, giving rise to a more chronic inflammatory state. In nearly every organ or tissue system, MMPs are involved at several levels of the inflammatory cascade. For instance, efficient migration and extravasation of leukocytes along chemotactic gradients to sites of infection are important for establishing effective immunity and MMPs have been shown to contribute to these functions. MMPs aid in establishing a chemotactic signal for recruitment of leukocytes and at the same time degrade ECM and junctional proteins, promoting leukocyte infiltration. Chemokines are immobilized mostly on the ECM or cell surface by binding to glycosaminoglycans and MMPs might contribute to the liberation of these molecules, delivering soluble effectors in the extracellular environment [8]. Paracellular movement of leukocytes is impeded by tight junctions and adherens junctions that occlude the intercellular cleft. Occludin and* zonula occludens*-1 (ZO-1), important components of the intercellular tight junctional complex, have been identified as substrates of MMPs [9–11]. In addition, vascular endothelium- (VE-) cadherin and E-cadherin, major components of the adherens junction, are known to be cleaved by MMP-7 and MMP-9 [12–14]. Importantly, MMPs are derived from the injured tissue as well as from the infiltrating immune cells. MMP-8 and MMP-9 are, for instance, stored in intracellular granules in neutrophils. Macrophages are on the other hand important in attenuating the acute immune response. Here, MMPs contribute by removing the chemotaxis of neutrophils and by inhibiting T-cell proliferation and function [4, 5].

At a second level, MMPs regulate the availability and activity of inflammatory mediators, including cytokines and chemokines. Whereas the proinflammatory tumor necrosis factor-alpha (TNF*α*) is generally activated by MMPs, a dual role has been proposed with respect to interleukin-1-beta (IL1*β*) activity [15, 16].* Vice versa*, several cytokines are implicated in the (up)regulation of MMPs. As such, TNF*α*, IL1*β*, and transforming growth factor-beta (TGF*β*) are implicated in the upregulation of MMP-1, MMP-3, and MMP-9 via the nuclear factor kappa-B (NF*κ*B) transcription factor, thereby creating a positive feedback loop [17–19].

Finally, MMPs can trigger a specialized form of programmed cell death termed anoikis that is induced by cells detaching from the surrounding ECM by interrupting cell-cell and cell-matrix interactions [20].

A thorough discussion of isolated MMPs' contribution to inflammation falls beyond the scope of this review but is excellently reviewed by others [4, 21, 22]. There are however some aspects that we would wish to highlight here. A first and very relevant aspect of MMPs in view of this review is the activation kinetics of MMPs. Some MMPs, for example, MT-MMPs and the downstream MMP-2, are believed to be constitutively active, although their activity can still be enhanced in inflammatory conditions [23]. Oppositely, other MMPs such as MMP-9 are only induced and activated under conditions of immune activation and are normally associated with activated leukocytes, macrophages, and endothelium [2]. Being dependent on the presence of proinflammatory cytokines and a cascade of cleavage events by upstream proteases (other MMPs, plasmin), activation of MMPs is considered to be a slightly delayed (nevertheless acute) event in the inflammatory response [1, 23]. However, as we will discuss later, removal of the prodomain is not always required and MMPs may be acting at a faster time scale in such conditions. Second, the mode of activation largely determines the site of MMP activity. Being dependent on membrane-bound MT1- and MT3-MMPs for its activation, MMP-2, for example, is generally considered to be spatially constrained, whereas other MMPs like MMP-9 are released in the extracellular space and diffuse to more remote sites. Therefore, secreted MMPs are presumed to cause more widespread damage [23]. In addition, as outlined below, MMPs are now also known to cleave intracellular substrates, unlocking a new level of complexity with respect to their role in inflammation. In the following sections we will discuss their action on connexin (Cx) channel function. These channels too have been identified as important contributors to the inflammatory process (see later); however, only a small number of papers suggested a link between Cxs and MMPs. Their interaction at the functional level therefore remains poorly understood. Here, we try to explain how an MMP-Cx interaction may mechanistically alter channel function and contribute to acute inflammation.

## 2. Connexin Channels: Gap Junctions and Hemichannels

Cxs are a family of transmembrane proteins with molecular weights (MW) varying from 26 to 60 kDa on which the current nomenclature is based (e.g., Cx43 has a MW of ~43 kDa). Cxs form two kinds of channels, namely, gap junctions (GJs) and hemichannels (HCs). GJs mediate the direct diffusion of ions and molecules with MWs up to 1.5–2 kDa, including inositol 1,4,5 trisphosphate, cyclic nucleotides, and energy molecules such as glucose and ATP [24], thereby contributing to the coordination of cell function in several organs and tissues. GJ channel-mediated intercellular communication (GJIC) is, for instance, implicated in the communication of electrical signals between cardiomyocytes, coordinating cardiac pump function. GJIC between smooth muscle cells coordinates, for example, bladder and uterus function [25, 26]. GJs also pass signaling molecules to mediate the propagation of intercellular Ca^2+^ waves in various tissues and organs [27], they provide metabolic coupling between liver cells or astrocytes [28–30] and contribute to the exchange of bone modulating molecules [31]. However, on the downside, GJs also spread cell death signals to neighboring cells, thereby contributing to tissue/organ damage in pathology [32]. Half GJ channels that arise from the hexameric assembly of different Cx subunits can be present in the plasma membrane both as GJ precursors, called connexons, or as nonjunctional, functional channels, known as HCs. For a long time, it was thought that the only reason for a HC to open was related to their incorporation into a GJ channel. Uncontrolled HC opening was presumed to lead to membrane depolarization and depletion of essential molecules from the cytoplasm, ultimately resulting in cell dysfunction and possibly cell death. The first evidence of functional HCs arose from* in vitro* work using Cx46 expression in* Xenopus laevis* oocytes, indicating that HC opening resulted not only in dye uptake, but also in cell depolarization and cell death [33]. Research over the past decades has however identified numerous scenarios in which HCs are activated to open, thereby contributing to paracrine signaling through the release of ATP [34], glutamate [35], glutathione [36], NAD^+^ [37], and prostaglandins [38, 39]. HC-mediated ATP release, for instance, functions as a paracrine signal in the propagation of intercellular Ca^2+^ waves [27, 40]. Evidence is accruing that HCs may contribute to physiological functions such as “center-surround” antagonism in the retina [37, 41], osteogenesis [31, 42], regulation of vascular permeability [43], central chemoreception [44], and atherosclerotic plaque formation [45]. However, HCs also have an established role in pathological conditions associated with inflammation which has been particularly well-documented in the brain [46–54]. Finally, both HCs and GJs are important in the induction as well as the propagation of cell death [55].

In this review we will focus on the actions of MMPs on GJs and HCs formed by Cx43, which is ubiquitously present in a large array of cells and tissues in the human body [56]. Furthermore, Cx43 is the isoform that has been characterized in great detail in terms of intramolecular gating mechanisms as well as its role in inflammation at the functional level.

## 3. MMPs' Impact on Connexin Expression and Channel Function

### 3.1. Intracellular Action of MMPs

The only paper thus far indicating that Cx43 is a target for MMPs, more specifically MMP-7, has documented cleavage of the intracellularly located C-terminal domain [7]. Indeed, as outlined above, MMP activity is not confined to the extracellular space and substrates are much more diverse than just matrix proteins. At present, intracellular targets such as intracellular matrix proteins, enzymes, and molecular chaperones regulating transcription and translation are well known to be part of the MMP substrate repertoire. Multiple MMPs, including MMP-1, MMP-2, MMP-3, MMP-7, MMP-8, MMP-9, MMP-13, MMP-26, and MT1,3-MMP, have been shown to process intracellular proteins, indicating that intracellular activity of MMPs is not confined to one particular class of MMPs [3]. MMP-3 is, for example, activated in dopaminergic neurons by the apoptosis inducer BH4 and acts upstream of caspase-3, indirectly contributing to the cleavage and activation of this apoptotic mediator [57]. High throughput degradomics has furthermore identified a myriad of intracellular matrix proteins as substrates of MMP-9, many of which are linked to different autoimmune diseases. Such data indicate that MMP-9 may have an immune-regulatory function, removing toxic molecules that are released upon cell death, but also generating substrates for autoantigens [58]. Finally, intracellular activity of MMP-2 has been confirmed in fast twitch type II muscle fibers with protease activity being dependent on physical exercise [59]; MMP-2's specific role is however unknown in these cells. In platelets, intracellular activity of MMP-2 contributes to platelet aggregation [60, 61].

Intracellular activation of MMPs may be achieved by intracellular proteases that separate the prodomain from the catalytic domain. The Golgi-associated prohormone convertase furin, for instance, activates MMP-11 by cleavage at the Arg-X-Arg-X-Lys-Arg sequence. This recognition motif has also been identified in MT1-, MT2-, MT3-, MT4-, and MT5-MMPs and in MMP-23, while the similar Arg-X-X-Arg and Lys-X-X-Arg sequences have been found in all MMPs, except MMP-7 and MMP-12 [2, 3]. Other candidate mechanisms for intracellular proteolytic processing of MMPs include serine proteases, caspases, upstream intracellular MMPs, and autolytic cleavage [2, 3]. Trypsin-2 has, for instance, been shown to activate MMP-9 inside intracellular vesicles of epithelial cancer cells, thereby determining the aggressive and invasive character of these cells [62]. Intracellular MMP activation does not always require removal of the pro-domain. Reactive oxygen (ROS, e.g., peroxynitrite) and nitrogen (RNS) species that reassociated with oxidative stress may interact with the Cys-thiol group and disrupt the interaction with Zn^2+^, leading to autocatalytic activation while the full-length pro-MMP remains intact. S-nitrosylation and S-glutathionylation have been shown to activate MMP-1, MMP-2, MMP-8, and MMP-9 [2, 3, 63]. MMP-2, MMP-7, MMP-8, and MMP-9 activity have been furthermore shown to be dependent on ROS levels with low levels activating the proteases and high levels preventing protease activity. At the same time, ROS may also alter the structure and binding affinity of TIMPs, resulting in lower affinity and dissociation from the MMPs [64]. Alternatively, oxidative stress has been shown to promote activation of an alternate promoter located within the first intron of the MMP-2 gene, rendering an intracellularly active N-terminal truncated MMP-2 isoform that lacks the secretory sequence and the inhibitory prodomain region [65]. Intracellular activity of MMPs may be further facilitated by alternative splicing that renders MMP proteins lacking the secretory signal peptide [2, 3, 66, 67]. Finally, it has been proposed that intracellular activation of MMPs can be achieved by proteins belonging to the SIBLING (small integrin-binding ligand N-linked glycoprotein) family. BSP (bone sialoprotein), OSP (osteopontin), and DSP1 (dentin matrix protein 1), all upregulated in different types of cancer, may, respectively, bind and activate MMP-2, MMP-9, and MMP-3 without removing the prodomain, but by inducing conformational changes in the protease [68].

### 3.2. MMP Activity Correlates with Altered Connexin Expression

Evidence for the proteolytic processing of Cx proteins by MMPs is mostly derived from the heart where Cx40, Cx43, and Cx45 are expressed in a site-specific manner with Cx43 present in atrial tissue and being most prevalent in the ventricles. Cx40 and Cx45 are present in SA and AV nodes and in atrial and ventricular tissue, respectively [69, 70]. In the heart, GJs mediate electrical coupling and direct cell-to-cell transfer of chemical and metabolic signals. Consequently, changes of GJ properties are collectively known to contribute to myocardial infarction injury and arrhythmogenesis. Intravenous injection of the proinflammatory cytokine TNF*α* increased MMP-2 levels in mouse atrial tissue which was correlated with a decrease in Cx40 expression [71]. In canine ventricular tissue, Cx43 expression became progressively weaker and disordered with the duration of ventricular fibrillation. At the same time, a decline in TIMP-2 levels and increase in MMP-2/TIMP-2 ratio were observed [72]. Cardiac pressure overload in TIMP-2 knockout mice was associated with increased levels of MMP-9 and MMP-14, leading to a decreased expression of the endocardial Cx37 as well as Cx43, thereby exacerbating cardiac dysfunction [73]. In cardiac fibroblasts, expression of both MMP-2 and MMP-9 was increased and associated with a concomitant decrease in Cx43 expression after activation of endothelin receptors [74]. Finally, elevated levels of homocysteine, a sulfur-containing nonprotein amino acid and a strong inducer of oxidative stress, activated MMP-9 in mouse ventricular myocytes which led to Cx43 mitochondrial translocation and degradation [75]. A link between Cx expression and MMP activation has also been described in extracardiac tissues, for example, in the retinal endothelial cells, where hyperglycemia increased mitochondrial MMP-2 activity, leading to a downregulation of Cx43 as well as the induction of apoptotic cell death. Treatment with MMP-2 small interfering RNAs prevented the decrease in Cx43 and protected against apoptosis [76]. Oppositely, in hyperglycemic kidneys, ROS activated MMP-9 which was accompanied by an upregulation of Cx40 and Cx43 [77].

**Figure 1:**
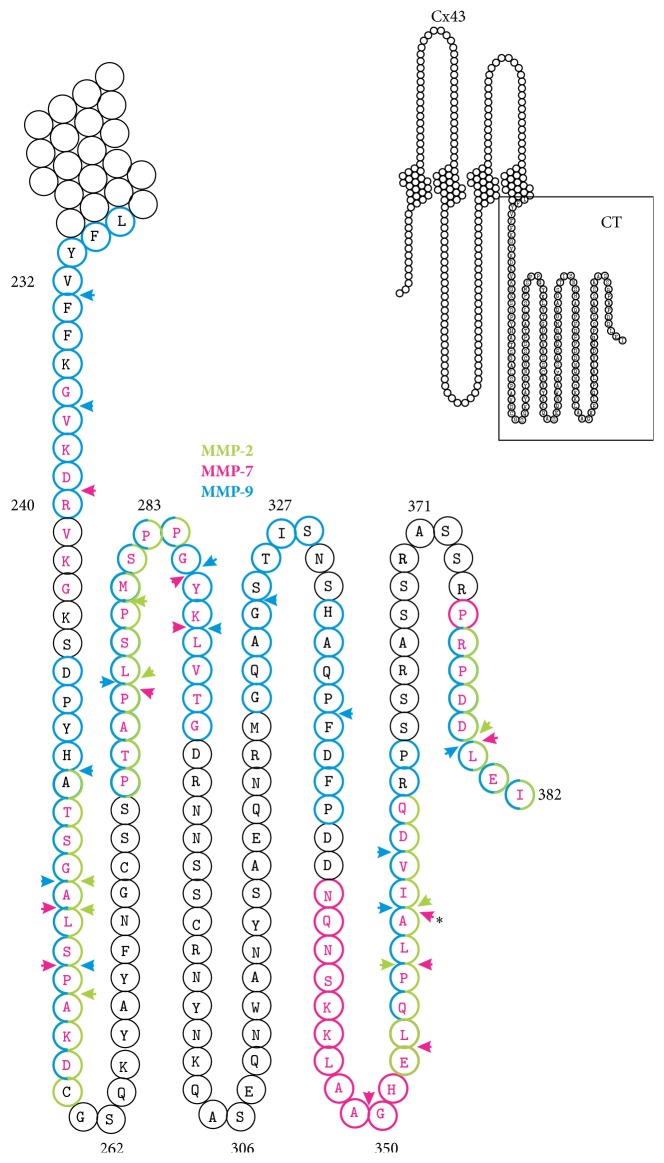
Predicted MMP cleavage sites of the Cx43 C-terminal domain.* In silico* analysis using PROSPER and SitePrediction reveals several potential cleavage sites of MMP-2 (green circles), MMP-7 (pink letters), and MMP-9 (blue circles) in the human Cx43 C-terminal domain. MMP target domains are 8 amino acids in length (P4-P3-P2-P1-P1′-P2′-P3′-P4′) with the actual MMP cleavage site (between P1 and P1′) indicated by the arrowhead. In addition, we include one MMP-7 target site published in [7] that is not predicted by* in silico* analysis using PROSPER or SitePredict (indicated by the asterisk). Inset depicts the full length topology of Cx43.

Despite correlation between increased MMP expression and decreased Cx expression levels, none of the papers referred to above unequivocally demonstrated a direct role of MMP proteolytic activity in regulating Cx expression. In fact, very few papers have provided such direct evidence. Wu et al. have recently indicated that, in rat H9C2 cardiomyocytes, hypoxia decreased the total Cx43 protein level by ~30–50% in a MEK/ERK MAPK-dependent and MMP-9-dependent manner. The Zn^2+^ chelating compound doxycycline largely prevented the decline in Cx43 [78]. Doxycycline is best known as a broad-spectrum antibiotic tetracycline but also acts as a broad-spectrum MMP inhibitor at subantimicrobial doses. Furthermore, the most straightforward evidence for the proteolytic processing of Cx43 by MMPs was provided by Lindsey et al. [7]. In postmyocardial infarction heart sections, Cx43 staining was decreased while cardiomyocyte MMP-7 levels were significantly increased. Accordingly, Cx43 downregulation was not observed in MMP-7 knockout mice. Further evaluation by surface plasmon resonance (SPR) protein binding studies demonstrated a direct and specific interaction between Cx43 and MMP-7. Importantly, decreased Cx43 detection levels were observed when using an antibody targeting the last 10 C-terminal amino acids (373–382), but not when using an antibody targeting amino acids (252–270) that are located more upstream in the C-terminal domain. This argued in favor for the proteolytic cleavage of Cx43 C-terminal amino acids, rather than an overall decrease in Cx43 expression levels.* In silico* analysis indeed revealed two sites with sequence homology to known MMP-7 cleavage sequences within the Cx43 C-terminal domain: ^341^NQNAKKVAAGHELQPLAIVD^360^ shows similarity with the MMP-7 cleavage sequence GPQAIAGQ; ^375^PRPDDLEI^382^ shows similarity with the MMP-7 cleavage sequence PPEELKFQ [7] (Figure 1).

We performed further* in silico* analysis of possible MMP cleavage sites in the human Cx43 C-terminal domain using PROSPER (http://lightning.med.monash.edu.au/PROSPER/) [79] and SitePrediction (http://www.dmbr.ugent.be/prx/bioit2-public/SitePrediction/) [80]. SitePrediction uses known datasets available in literature to identify possible cleavage sites in a given amino acid sequence. It combines similarity scores of the candidate sequence with known cleavage sites, with frequency scores that indicate whether amino acids of the candidate sequence are likely to occur at the cleavage domain recognized by a specific protease [80]. SitePrediction allows predicting of cleavage sites of numerous proteases, including MMP-1, MMP-2, MMP-3, MMP-7, MMP-8, MMP-9, MMP-12, and MMP-13. Of these, only MMP-2, MMP-7, and MMP-9 have been described in the context of altered Cx43 expression/function and we therefore chose to focus only on these three MMPs. SitePrediction identifies numerous potential cleavage sites in the Cx43 C-terminal domain that have 95% specificity (the chance that the identified site is an actual cleavage site) (Table 1). Analysis using PROSPER seems more stringent as compared to SitePrediction as only one cleavage domain is identified for MMP-2 and no candidate sites for MMP-7 are recognized (Table 2). Like SitePrediction, PROSPER identifies protease substrates and their cleavage sites, using info available in the peptidase database MEROPS. It furthermore combines a number of complementary sequence and structural features, including local amino acid sequence profile, predicted secondary structure, solvent accessibility, and natively disordered region, as well as some global sequence features, for predicting cleavage sites of protease substrates [79]. The resulting probability score (that describes the quantitative cleavage probability for each cleavage site) contains a confidence in the prediction and only cleavage sites with a predicted cleavage probability score greater than 0.8 are listed. Only MMP-2, MMP-7, MMP-9, and MMP-3 are available in PROSPER [79] and we again focused on MMP-2, MMP-7, and MMP-9 for identifying potential cleavage sites in the Cx43 protein. Figure 1 summarizes the potential target sites of MMP-2, MMP-7, and MMP-9 in the Cx43 C-terminal domain as identified by PROSPER and SitePrediction. We stress, however, that* in silico* analysis is predictive and requires further experimental validation. For instance, although an interaction between Cx43 and MMP-7 has been confirmed by SPR [7], PROSPER did not identify MMP-7 as a protease that cleaves Cx43. SitePrediction does, but the identified domains do not correspond with those described by Lindsey et al. [7]. In addition, one of the sites identified in [7] (Figure 1, indicated by *∗*) has an average score of 0.005 and a specificity far below 95% in SitePrediction. Oppositely, both PROSPER and SitePrediction identified MMP-2 as a potential candidate, but MMP-2 failed to bind Cx43 in SPR studies [7].

Based on the data presented by Lindsey et al. [7] and those obtained with* in silico* analysis demonstrating cleavage of the C-terminal domain, a careful reevaluation of previous studies reporting Cx43 downregulation should perhaps be considered. Indeed, most commercially available antibodies against Cx43 target the C-terminal domain, but unfortunately, epitopes are not always mentioned in studies claiming Cx43 downregulation.

### 3.3. Functional Consequences of C-Terminal Cx43 MMP-Cleavage at the Channel Level

**Figure 2:**
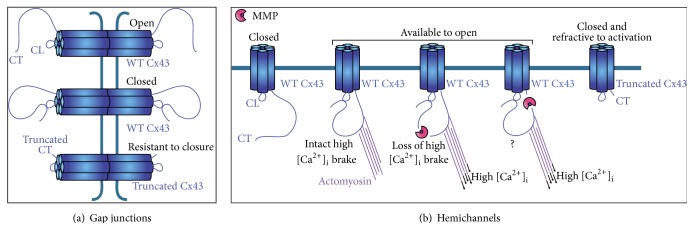
Cx43 channel gating by CT-CL interactions and possible effects of Cx43 C-terminal cleavage on hemichannel function. The Cx43 C-terminal domain is intricately involved in gating of both HCs and GJ channels. (a) In normal conditions, GJ channels are open, with the C-terminal domains not interacting with the CLs. GJ closure occurs when the CT binds the CL (ball-and-chain closure). In GJ channels composed of CT-truncated Cx43, closure via the ball-and-chain mechanism cannot occur and GJ channels remain open. (b) An intramolecular CT-CL interaction has been proposed to bring Cx43HCs in the “available to open” state whereas in the absence of such interaction, HCs remain closed. HC closure at above 500 nM [Ca^2+^]_i_ is mediated by cytoskeletal contractions that dislocate the C-terminal domain from the CL and act as a brake on HC opening. Such CT-CL interaction cannot take place in HCs consisting of C-terminally truncated Cx43, making them refractive for activation. MMP cleavage of Cx43HCs in the “available to open” state will result in a C-terminal peptide that is bound to the CL. This will cause loss of the high [Ca^2+^]_i_ brake when the cleavage site is located downstream of the Cx43-actomyosin interaction site. When the MMP cleavage site is located N-terminally of this actomyosin linker domain, the outcome is less clear. In principle, actomyosin contraction may remove the CT peptide from the CL, but a residual interaction of the CL with more upstream sequences may keep the HC in an “available to open” state. Identification of the actomyosin interaction domain within the Cx43 C-terminal domain responsible for mediating the high [Ca^2+^]_i_ brake on HC opening will resolve these uncertainties.

Cx43 is by far best characterized in terms of the role of its C-terminal domain in modulating channel function. This domain comprises amino acids 232–382 and is the primary interaction domain of Cx-associated partner proteins like ZO-1, tubulin, microtubules, and caveolins that may regulate protein trafficking and function [81–83]. Additionally, it is the prime target for posttranslational modifications such as S-nitrosylation [84] and phosphorylation [85]. Under both basal and stimulated conditions, Cx channel activity appears to be regulated by ongoing phosphorylation-dephosphorylation events. However, much of the details on how Cx phosphorylation can determine the activity state of HCs and GJs still remains to be resolved [85]. Interestingly, certain kinases, including pH-dependent kinases [86], act on the Cx43 C-terminal domain, a molecular hotspot for the control of GJ and HC activity. Phosphorylation of the CT tail may add negative charges at this site, potentiating an interaction between the CT and the second half of the cytoplasmic loop (CL), termed the L2 region (AAs 119–144). As such, the L2 region, which contains a stretch of positively charged AAs, serves as a receptor domain for the CT [87, 88]. This intramolecular CT-CL interaction has been proposed to act as a ball-and-chain mechanism mediating the closure of GJ channels, for instance, during voltage gating and chemical gating of GJ channels by intracellular acidification [89–95] (Figure 2(a)). The CT-CL interaction is expected to induce a change in channel conformation that brings the GJs in a* closed* state [96]. Strikingly, for Cx43HCs, such CT-CL interaction is essential for HC* opening*. This was suggested for the first time in studies investigating the bimodal response of Cx43HCs to an increase in [Ca^2+^]_i_. A moderate increase in [Ca^2+^]_i_ up to 500 nM strongly promotes Cx43HC opening while this effect disappears with larger [Ca^2+^]_i_ elevations to the micromolar level that tend to close the HCs [97–99]. Mechanistically, Ca^2+^-activation of Cx43HCs is mediated by calmodulin-dependent signaling [98] and is dependent on a CT-CL interaction [100] that brings the HCs in the “available to open” state [96] (Figure 2(b)). Importantly, CT-CL interaction is a necessary condition for HC opening, but the actual opening is triggered by membrane depolarization or moderate (<500 nM) elevation of [Ca^2+^]_i_. HC closure at above 500 nM [Ca^2+^]_i_ is mediated by cytoskeletal contractions that pull the C-terminal domain away from the CL [96, 101] (Figure 2(b)). The latter system acts as a brake on HC opening and is operational under physiological conditions presumably to prevent the detrimental effect of uncontrolled opening of this large conductance channel. The dependence of Cx43HC opening on a CT-CL interaction stands in stark contrast to the fact that such interaction results in closure of GJs [102]. At the molecular level, it is still uncertain why and how GJs and HCs are differentially modulated by a CT-CL interaction. Nonjunctional HCs (closed) may adopt different conformations as compared to those incorporated into GJs (open). Interactions between subunits during docking of two HCs may indeed result in conformational changes of the Cx protein, thereby altering gating properties. Another element that may contribute is the fact that HCs and GJ channels are differentially located in different plasma membrane domains with different properties such as lipid rafts [48].

Like most transmembrane proteins, Cxs are cotranslationally integrated into the rough endoplasmic reticulum (ER) membrane where they adopt their native transmembrane configuration [103, 104]. The subsequent oligomerization of Cx proteins into HCs starts in the ER, progressing to the trans-Golgi network [103, 104]. After leaving the ER-Golgi intermediate compartment, Cxs then transit through the cis- and trans-Golgi network before being shuttled to the PM [56, 105]. Some data indicate that Cx43 is transiently phosphorylated early in the secretory pathway [106], suggesting that the CT is exposed and available for interaction with modifying proteins during transit from the ER to the Golgi network and plasma membrane. Thus, in principle, intracellular cleavage of the Cx43 C-terminal domain may not only occur in plasma membrane Cx43 channels, but also in channels that are “en route” to the plasma membrane. Work with CT-truncated Cx43 mutants has repetitively shown that CT-truncated proteins are present at the plasma membrane of mammalian cells [93, 107–109]. Consequently, also following MMP cleavage, channels harboring truncated Cx43 will be present at the plasma membrane. Note though that* in vivo* data describing the trafficking behavior of disease-associated Cx43 mutations giving rise to preliminary CT-truncated Cx43 indicates that these truncated proteins are not inserted in the plasma membrane [110–113].

GJs have generally been shown to remain functional when composed of truncated Cx43. This has been confirmed by dye coupling studies for Cx43^M239stop^ [114] and Cx43^D378stop^ [115] mutants. Dual patch clamp studies have revealed similar results at the macroscopic level for Cx43^D378stop^ [115] and Cx43^K258stop^. Yet, single channel analysis of the latter revealed that mean open time was prolonged and transitions to intermediate, residual open states were lost. This was demonstrated both in cardiomyocytes [107] and astrocytes [116]. GJs formed by truncated Cx43 were furthermore found to be resistant to closure upon intracellular acidosis [117]. Opposite to GJs, HCs containing CT-truncated Cx43 become refractive to activation in response to membrane depolarization [34] and increased [Ca^2+^]_i_ (Cx43^M239stop^ [100]) or by omission of extracellular Ca^2+^ form the culture medium (Cx43^M239stop^ [114]), most likely because the CT-CL interaction that is necessary for HC opening is lost. Opposite results exist for HCs formed of truncated Cx43 (Cx43^M258stop^) that still open in zero Ca^2+^ conditions [116]. As the mechanism of Cx43HC opening by depletion of extracellular Ca^2+^ conditions remains to be fully elucidated, it is uncertain whether the short, remaining CT stretch in the Cx43^M258stop^ versus the Cx43^M239stop^ can be responsible for this differential outcome.

C-terminal Cx43 cleavage by MMPs not only results in a truncated protein with compromised CT-CL interaction capabilities but also renders small, free endogenous C-terminal peptides that have potential to alter channel function in their own right [7]. This is exemplified by application of exogenous peptides that mimic the last 9 amino acids of the Cx43 protein (RPRPDDLEI). ACT1, developed by Gourdie and coworkers, is an example of such a peptide that is N-terminally linked to an antennapedia cell-penetrating peptide [118]. ACT1 interferes with the binding of ZO-1 to the C-terminal domain, thereby sequestering undocked Cx43 connexons into GJs, enhancing GJ aggregation and potentiating GJIC, without stimulating Cx43 expression [118, 119]. ACT1 is currently under investigation as a novel therapeutic in wound healing [120–123] and may also be applicable as an antiarrhythmogenic compound [124] as well as a tumor suppressor [125]. The ACT1 peptide was only found effective as a HC inhibitor in confluent cell monolayers, but not in semiconfluent cells. It was therefore hypothesized that ACT1 inhibits HCs only because more HCs are incorporated into GJ channels [119]. However, a slightly different picture has been proposed by Ponsaerts et al. [100]. In this collaborative work, we used the very same C-terminal peptide (RPRPDDLEI) but linked to the TAT translocation sequence (derived from the HIV-1 virus), to investigate HC gating by [Ca^2+^]_i_ changes in subconfluent cell cultures. Here, addition of the “TAT-CT” peptide prevented HC closure at high [Ca^2+^]_i_ by binding to the CL, thereby mimicking the endogenous CT-CL interaction. Exogenously added TAT-CT peptide is thus able to substitute for the endogenous CT sequence. In line with this, TAT-CT restored HC activity of C-terminally truncated Cx43 (Cx43^M239stop^), while not affecting HC activation by modest (<500 nM) [Ca^2+^]_i_. The lack of TAT-CT effect on closed Cx43HC in resting conditions points to a scenario whereby CT-CL interaction is a necessary condition for HC opening triggered by stimuli such as strong membrane depolarization or moderate (<500 nM) [Ca^2+^]_i_ elevation. Some additional activation steps may be necessary that first expose the CL domain for subsequent binding of the CT [96]. In this context, ZO-1 did not seem to play a role in the modulation of HC function by TAT-CT since a TAT-CT version lacking the last isoleucine residue, essential for interaction with ZO-1, was fully capable of alleviating closure by high [Ca^2+^]_i_ and of restoring the activity of Cx43^M239stop^ HCs [100]. This was later confirmed in Cx43^D378stop^ adult hearts where Cx43 and ZO-1 still normally colocalized at the intercalated disk despite the absence of the 5 last C-terminal amino acids [115]. As a consequence of these considerations, the peptide resulting from cleavage of the Cx43 C-terminal domain at ^375^PRPDDLEI^382^ as has been described for MMP-7 [7] and suggested for MMP-9 (Figure 1), may thus prevent pathological closure of GJs while promoting HC opening at high [Ca^2+^]_i_. On a longer time scale (hours/days), HC opening may be dampened as suggested by the work with ACT1 peptide.

Finally, forced expression of Cx43 C-terminal fragments has additionally revealed their translocation to the nucleus where they act to inhibit cell growth and abrogate differentiation [126–129]. Thus, MMP generated CT fragments may potentially act via nuclear signaling as well.

Intriguingly, endogenous, cytoplasmic C-terminal fragments of Cx43, about 20 kDa in size have been observed in cultured murine and hamster cells and tumor cells and in cardiac cells subjected to ischemia [130–134]. These naturally occurring fragments have been suggested to result from internal translation of the* GJA1* gene transcript [133] since their occurrence could not be prevented by the MMP protease inhibitor EGTA-complete(R) and the serine protease inhibitor phenylmethylsulfonyl fluoride (PMSF) [131]. However, it looks less convincing that addition of protease inhibitors in the lysis buffer would prevent the occurrence of previously MMP-cleaved fragments in the lysate. In another paper, cleavage was excluded as a possible source of these fragments because detection with an N-terminal Cx43 antibody could only reveal full-length protein but not truncated Cx43 [132]. Obviously, posttranscriptional control of Cx43 expression at the mRNA level may explain some of these results but we propose to carefully consider the alternative option of cleavage by proteases. Indeed, in most cases, these C-terminal 20 kDa fragments mainly remain at the observational level and their origin is not discussed. Particularly in conditions known to induce MMP activity such as ischemia, protease activity may well contribute to the cellular production of these fragments.

## 4. Implications of Altered Connexin Channel Function in Inflammation

Several studies specifically implicate Cx43HCs in various injuries and inflammatory pathways. Some evidence comes from the cardiac system [48, 135, 136] but most evidence derives from the central nervous system where increased Cx43 expression has been observed following stroke, epilepsy, optic nerve damage, spinal cord injury, amyloid plaque formation, and MS and where Cx43HC opening leads to increased damage via the inflammatory response [47, 52, 137–144]. Cx43HC opening in inflammatory conditions is known to be mediated by advanced glycation end-products (AGEs) [145], oxidative stress [84, 145], and proinflammatory cytokines [146, 147]. Almost simultaneously, Retamal et al. [146] and Morita et al. [147] were the first to show that exposure of cultured astrocytes to (microglia-derived) proinflammatory cytokines such as IL1*β* and TNF*α* stimulated Cx43HC opening. This was mediated by the activation of p38 MAPK and increase in NOS activity and NO production [146]. Acute opening of astroglial Cx43HCs by IL1*α*/*β* was later confirmed using live brain slices from mice harboring* S. aureus* induced abscesses [148].

Upon opening, Cx43HCs form a well-known pathway for the release of ATP which is driven by a large concentration gradient between the intra- and extracellular compartment [34, 149, 150]. This purinergic messenger is a crucial factor in establishing a chemotactic signal for infiltrating polymorphonuclear neutrophils [151–154]. Modulation of Cx43HCs and inhibition of ATP release indeed correlate with reduced tissue invasion of neutrophils [155]. Neutrophils on their part also release ATP by means of Cx43HCs, further contributing to the progression of the inflammatory response [156, 157]. In addition, Cx43HC-mediated ATP release can actively contribute to the activation of the NLRP3 inflammasome, a protein complex that serves to sense pathogen- and danger-associated molecular patterns and is involved in IL1*β* and IL18 processing [158]. Using the endothelial cell line b.End5, Robertson and coworkers have furthermore indicated that ATP release induces the expression of Toll-like receptor-2 (TLR2) and production of IL6 upon infection with* S. epidermidis*. In turn, TLR2 activation gave rise to a further upregulation of Cx43 expression, albeit it had no impact on actual Cx43HC opening [159]. Not only ATP but also other active compounds released by HCs might contribute to progression of inflammation. For instance, inhibition of glutamate release from activated microglia has proven beneficial in the outcome of spinal cord injury, reducing glial scar formation and increasing expression of growth factors [140]. Recently, Cx43HCs were also found to play a role in neuropathic pain. Here, Cx43 expression was upregulated in spinal cord astrocytes following chronic constriction injury and subsequent Cx43HC opening led to the release of the chemokine CXCL1 [160].

In short, aberrant Cx43HC-mediated signaling may promote acute inflammation. Intriguingly, all of the above-mentioned pathological conditions (stroke, epilepsy, bacterial infection, etc.) in which Cx43HCs contribute to inflammation are also associated with increased MMP activity [23, 161–166]. In the following paragraphs we set out a hypothesis of how proteolytic cleavage of MMPs can contribute to inflammation.

Interestingly, knockout of the MMPs that have been associated with reduced Cx43 expression/function (i.e., MMP-2, MMP-7, and MMP-9) leads to altered chemotaxis, attenuated leukocyte influx, and reduced cell death [8, 167–173]. However, an equal amount of papers describes impaired repair, increased leukocyte load, and an aggravated inflammatory response upon knockout of these MMPs [13, 174–176].

As described above, an important feature of the MMPs is their minor latency of activation, with oxidative stress and/or expression of proinflammatory cytokines generally preceding MMP activity. Interestingly, activation of Cx43HCs by the proinflammatory cytokines TNF*α*, IL1*β*, and IFN*γ* requires exposure times in the order of 9 h [177], 24 h [146], and 48 h [178]. These very slow actions may well reflect the involvement of MMPs. Additionally, MMP expression and activity depend on an increase of [Ca^2+^]_i_ triggered by proinflammatory cytokines [179]. This [Ca^2+^]_i_ increase will also activate Cx43HC opening when HCs are in the “available to open” state. Subsequent MMP-cleavage in the CT will generate a C-terminal peptide that is bound to the CL (Figure 2(b)). The consequent effect of proteolytic cleavage on HC function may depend on the position of the MMP cleavage site relative to the yet unidentified Cx43-actomyosin interaction domain(s) in the Cx43 C-terminal tail that mediates high [Ca^2+^]_i_-induced HC closure (“high [Ca^2+^]_i_ brake”). When cleavage occurs at a site that is situated C-terminally from the Cx43-actomyosin interaction domain, it is expected that the C-terminal peptide cannot be removed from the CL by cytoskeletal/actomyosin contractions (see Section 3.3). Thus, HCs remain in the “available to open” state (with effective opening triggered by membrane depolarization or [Ca^2+^]_i_ increase), but the high [Ca^2+^]_i_ brake disappears, thereby promoting HC opening in cells with pathologically high [Ca^2+^]_i_ (Figure 2(b)). Alternatively, when cleavage takes place at a site that lies upstream at the N-terminal side of the actomyosin linkage domain, the outcome is less clear. Theoretically, it is possible that cytoskeletal contractions remove the C-terminal peptide from the CL; however, multiple amino acid domains in the CT may participate in the CT-CL interaction. Indeed, Cx43 C-terminal amino acid domains 281–295, 299–304, 341–327, 342–348, and 360–382 have all been shown to engage in the intramolecular interactions necessary for ball-and-chain GJ channel gating [180, 181]. For Cx43HCs, at present, only the last 10 amino acids have been described to contribute to the CT-CL interaction necessary for promoting HC opening. However, as for GJs, it is to be expected that more upstream domains also participate in this intramolecular interaction. Identification of the actomyosin interaction site in the Cx43 C-terminal domain will allow us to further define the effects of MMP cleavage upstream (N-terminally) of the actomyosin linkage domain on HC function. For GJ channels, various proteins have been suggested to function as a linker between the cytoskeleton and the Cx43 C-terminal tail, including drebrin [182], cortactin [183], ezrin [184], Src [185], CIP85 [186], CCN3 [187], and ZO-1 [100, 188, 189]. For HCs, the latter can be excluded as the intermediate between the cytoskeleton and the Cx43 C-terminal domain [100]; however, whether the other candidate proteins, mentioned in the context of GJs, also interact with Cx43HCs remains to be confirmed.

Oxidative stress, that is tightly linked to the intracellular Ca^2+^ household [190] and to fast, intracellular MMP activation, has also been shown to promote Cx43HC opening within a time frame of several minutes [84, 191]. As described above, MMP cleavage may in this case further promote HC opening mediated by a loss of their high [Ca^2+^]_i_ brake. Interestingly, Cx43HCs themselves may subsequently act as an entry channel for ROS that may further activate MMPs and HC through a positive feedback loop [191].

Increased opening of Cx43HCs following proteolytic processing by MMPs would give rise to exaggerated ATP release and leukocyte infiltration, altogether aggravating the inflammatory response. Additionally, persistent loss of ATP and nutrients would also result in cellular energy deprivation and ultimately cell death. Furthermore, in the brain, elevated levels of glutamate in the cerebral interstitial fluid are known to be excitotoxic and so HC glutamate release by microglial cells would give rise to neurodegeneration. Although HC opening in inflammatory conditions is generally considered deleterious for the cell, a few reports indicate that HC opening may act protective, for example, by releasing signaling molecules that activate Src and ERK-mediated survival signals or by releasing prostaglandin E2 that protects against apoptosis [192–194].

Opposite to HCs, generally taken, GJIC is reduced in inflammatory conditions, as shown for proinflammatory cytokines, ATP, and oxidative stress [146, 195–200]. Such uncoupling has dual effects on the inflamed tissue; on the one hand, it may act as a protective mechanism that encapsulates the injured cells and functionally separates them from the surrounding healthy tissue. However, at the same time, uncoupling also impedes the supply of energy and nutrients that are necessary for tissue repair processes. Notably, contrasting reports that describe persisting functional GJIC in inflammation are available as well [159, 201, 202]. GJs have, for instance, been shown to propagate oxidative stress and bystander cell death [32, 203]. Additionally, coupled cells are able to share antigens with and trigger a response in cytotoxic T-lymphocytes [204]. Finally, GJs have been suggested to mediate the propagation of NF*κ*B and MAPK activation from infected to noninfected cells, leading to IL8 production, also by the latter [201]. Interestingly, using a brain abscess model, Karpuk et al. [46] have indicated that the degree of GJIC inhibition is dependent on the distance to the lesion site and that coupling gradually increases with expanding distance from the lesion site. Despite the upregulation of Cx43 expression, uncoupling was observed at the third day after infection and persisted up to the 7th day, indicating a long lasting effect [46]. The mechanisms that mediate such persistent block of GJIC without affecting expression levels are currently unknown. Given the double role of GJIC, the differential outcome of uncoupling in inflammation, and uncertainties regarding the detailed mechanisms of uncoupling, it is very difficult to speculate on the functional outcome of proteolytic cleavage mediated by MMPs in terms of GJIC and its impact on inflammation.

Finally, CT truncation not only should be considered relevant in view of intramolecular gating mechanisms and single channel function but is also of utmost importance at the level of protein-protein interactions. As mentioned above, the Cx43 C-terminal domain contains interaction sites for, for example, ZO-1, occludin, claudin, tubulin, and the protooncogene Src [205, 206]. Deletion of the last 5 amino acids has no functional effects of the level of GJIC but nevertheless induces arrhythmogenesis due to aberrant, channel independent interactions of Cx43 with sodium and potassium channels [115]. The purinergic receptor P2Y1 is another example protein believed to interact with the C-terminal domain of Cx43 and its expression is reduced in Cx43 knockout animals [207, 208]. Via their interaction with proteins that contribute to tight and adherens junctions (e.g., ZO-1 and occludin), Cxs stabilize the junctional complex that is situated between epithelial and endothelial cells and impedes paracellular diffusion [209–212]. A reduction in Cx43 expression is often accompanied by a downregulation of the junctional proteins leading to compromised intercellular junctions. Importantly, destabilization of the junctions abrogates the barrier function of epithelial and endothelial cells and therefore facilitates the paracellular movement of leukocytes into the inflamed tissue. On the other hand, exposure of astrocytes to IL1*β* leads to a concomitant downregulation of Cx43 and upregulation of the tight junctional protein claudin-1. It is believed that the latter brings astrocytes closer together, reducing the extracellular space volume and forcing inflammatory molecules to move in a particular direction [198]. Notably, connexin protein interactions with junctional proteins also stabilize the GJ plaques [213, 214]. In addition, many of the Cx43 interaction partners, including ZO-1 and occludin, are also MMP substrates and their proteolytic cleavage is expected to induce GJ uncoupling. Yet, such matter is ought to be addressed in future studies.

## 5. Conclusions

Via their remodeling of ECM and intercellular junctions as well as by their proteolytic processing of cytokines, chemokines, and growth factors, MMPs importantly contribute to inflammation, a process in which they are known to have beneficial as well as detrimental functions. In this paper we specifically describe the regulation of Cx43 expression and channel function by the intracellular action of MMPs. Cx43 is the most prevalent building block of GJs and HCs, two types of channels intricately involved in tissue homeostasis as well as in acute inflammation. A handful of reports describe a link between altered expression of Cx43 on the one hand and elevated levels of MMP activation on the other.* In silico* analysis additionally demonstrates that MMPs are capable of mediating cleavage of the Cx43 C-terminal domain which is an important determinant of HC and GJ channel function. Such cleavage has also been directly demonstrated in cardiac tissue where it contributes to tissue damage following myocardial infarction. Unfortunately, until now, no studies have been performed that unequivocally demonstrate the direct impact of MMPs on channel function at the mechanistic level. We here explain the possible effects of C-terminal cleavage on Cx43 channel function, using available information that comes from work with Cx43 truncated mutants and studies with exogenous C-terminal peptides as a basis. However, in inflammatory conditions where the oligomerized Cx43 protein is cleaved in the plasma membrane instead of being exogenously expressed as a truncated mutant, the underlying mechanisms may be very different. In addition, much like MMPs, the contribution of GJs and HCs to the inflammatory process seems very diverse with inhibition of the channels resulting in a positive as well as a negative outcome, depending on the tissue, the trigger, and the timing. Therefore, future challenges will be to better understand the role of MMPs and Cx43 channels in inflammation and to gain detailed insight in the nature of MMP-Cx interactions as well as in the effects on channel function before gearing up to the therapeutic level.

## Figures and Tables

**Table 1:** Results of cleavage site prediction based on SitePrediction^*∗*^ *in silico* analysis.

Matrix-metalloprotease	Position	Segment	Average score	Specificity
MMP-2	357^#^	QPLA −/− IVDQ	476.396	>99%
	355	ELQP −/− LAIV	117.022	>99%
	277	PTAP −/− LSPM	155.854	>99%
	254	TSGA −/− LSPA	106.264	>99%
	256	ALSP −/− AKDC	26.467	>95%
	326	AGST −/− ISNS	25.335	>95%
	252	ATSG −/− ALSP	22.937	>95%
	280	PLSP −/− MSPP	19.688	>95%
	379	RPDD −/− LEI	18.873	>95%

MMP-7	355	ELQP −/− LAIV	29.767	>99%
	287	PGYK −/− LVTG	29.767	>99%
	352	AGHE −/− LQPL	5.998	>95%
	255	GALS −/− PAKD	5.152	>95%
	277	PTAP −/− LSPM	4.057	>95%
	379	RPDD −/− LEI	3.397	>95%
	253	TSGA −/− LSPS	3.242	>95%
	285	SPPG −/− YKLV	2.469	>95%
	349	KLAA −/− GHEL	2.431	>95%
	238	GVKD −/− RVKG	2.119	>95%

MMP-9	285^#^	SPPG −/− YKLV	47.023	>99%
	324	GQAG −/− STIS	35.498	>99%
	255	GALS −/− PAKD	13.580	>95%
	357^#^	QPLA −/− IVDQ	11.443	>95%
	252	ATSG −/− ALSP	11.129	>95%
	235	FFKG −/− VKDR	4.991	>95%

^*∗*^ http://www.dmbr.ugent.be/prx/bioit2-public/SitePrediction/; ^#^also identified by PROSPER.

**Table 2:** Results of cleavage site prediction based on PROSPER^*∗*^ *in silico* analysis.

Matrix-metalloprotease	Position	Segment	Probability score
MMP-2	357^#^	QPLA −/− IVDQ	1.06

MMP-9	357^#^	QPLA −/− IVDQ	1.21
	285^#^	SPPG −/− YKLV	1.10
	248	DPYH −/− ATTG	1.08
	231	LFYV −/− FFKG	1.08
	359	LAIV −/− DQRP	1.03
	287	PGYK −/− LVTG	1.01
	334	HAQP −/− FDFP	0.98
	277	PTAP −/− LSPM	0.97
	379	RPDD −/− LEI	0.96

^*∗*^ https://prosper.erc.monash.edu.au; ^#^also identified by SitePrediction.

## References

[1] Yong V. W. (2005). Metalloproteinases: mediators of pathology and regeneration in the CNS. *Nature Reviews Neuroscience*.

[2] Chow A. K., Cena J., Schulz R. (2007). Acute actions and novel targets of matrix metalloproteinases in the heart and vasculature. *British Journal of Pharmacology*.

[3] Cauwe B., Opdenakker G. (2010). Intracellular substrate cleavage: a novel dimension in the biochemistry, biology and pathology of matrix metalloproteinases. *Critical Reviews in Biochemistry and Molecular Biology*.

[4] Nissinen L., Kähäri V.-M. (2014). Matrix metalloproteinases in inflammation. *Biochimica et Biophysica Acta—General Subjects*.

[5] Khokha R., Murthy A., Weiss A. (2013). Metalloproteinases and their natural inhibitors in inflammation and immunity. *Nature Reviews Immunology*.

[6] Giannandrea M., Parks W. C. (2014). Diverse functions of matrix metalloproteinases during fibrosis. *Disease Models and Mechanisms*.

[7] Lindsey M. L., Escobar G. P., Mukherjee R. (2006). Matrix metalloproteinase-7 affects connexin-43 levels, electrical conduction, and survival after myocardial infarction. *Circulation*.

[8] Van Lint P., Libert C. (2007). Chemokine and cytokine processing by matrix metalloproteinases and its effect on leukocyte migration and inflammation. *Journal of Leukocyte Biology*.

[9] Schubert-Unkmeir A., Konrad C., Slanina H., Czapek F., Hebling S., Frosch M. (2010). *Neisseria meningitidis* induces brain microvascular endothelial cell detachment from the matrix and cleavage of occludin: a role for MMP-8. *PLoS Pathogens*.

[10] Reijerkerk A., Kooij G., Van Der Pol S. M. A., Khazen S., Dijkstra C. D., De Vries H. E. (2006). Diapedesis of monocytes is associated with MMP-mediated occludin disappearance in brain endothelial cells. *The FASEB Journal*.

[11] Lu D.-Y., Yu W.-H., Yeh W.-L. (2009). Hypoxia-induced matrix metalloproteinase-13 expression in astrocytes enhances permeability of brain endothelial cells. *Journal of Cellular Physiology*.

[12] Navaratna D., McGuire P. G., Menicucci G., Das A. (2007). Proteolytic degradation of VE-cadherin alters the blood-retinal barrier in diabetes. *Diabetes*.

[13] McGuire J. K., Li Q., Parks W. C. (2003). Matrilysin (matrix metalloproteinase-7) mediates E-cadherin ectodomain shedding in injured lung epithelium. *The American Journal of Pathology*.

[14] Ichikawa Y., Ishikawa T., Momiyama N. (2006). Matrilysin (MMP-7) degrades VE-cadherin and accelerates accumulation of beta-catenin in the nucleus of human umbilical vein endothelial cells. *Oncology Reports*.

[15] McQuibban G. A., Gong J.-H., Tam E. M., McCulloch C. A. G., Clark-Lewis I., Overall C. M. (2000). Inflammation dampened by gelatinase a cleavage of monocyte chemoattractant protein-3. *Science*.

[16] Ito A., Mukaiyama A., Itoh Y. (1996). Degradation of interleukin 1beta by matrix metalloproteinases. *The Journal of Biological Chemistry*.

[17] Han Y.-P., Tuan T.-L., Hughes M., Wu H., Garner W. L. (2001). Transforming growth factor-*β*- and tumor necrosis factor-*α* -mediated induction and proteolytic activation of MMP-9 in human skin. *Journal of Biological Chemistry*.

[18] Sakai T., Kambe F., Mitsuyama H. (2001). Tumor necrosis factor *α* induces expression of genes for matrix degradation in human chondrocyte-like HCS-2/8 cells through activation of NF-*κ*B: abrogation of the tumor necrosis factor *α* effect by proteasome inhibitors. *Journal of Bone and Mineral Research*.

[19] Vincenti M. P., Coon C. I., Brinckerhoff C. E. (1998). Nuclear factor *κ*B/p50 activates an element in the distal matrix metalloproteinase 1 promoter in interleukin-1*β*-stimulated synovial fibroblasts. *Arthritis & Rheumatism*.

[20] Mannello F., Luchetti F., Falcieri E., Papa S. (2005). Multiple roles of matrix metalloproteinases during apoptosis. *Apoptosis*.

[21] Vandenbroucke R. E., Libert C. (2014). Is there new hope for therapeutic matrix metalloproteinase inhibition?. *Nature Reviews Drug Discovery*.

[22] Le N. T. V., Xue M., Castelnoble L. A., Jackson C. J. (2007). The dual personalities of matrix metalloproteinases in inflammation. *Frontiers in Bioscience*.

[23] Rosenberg G. A. (2002). Matrix metalloproteinases in neuroinflammation. *Glia*.

[24] Alexander D. B., Goldberg G. S. (2003). Transfer of biologically important molecules between cells through gap junction channels. *Current Medicinal Chemistry*.

[25] Neuhaus J., Weimann A., Stolzenburg J.-U., Wolburg H., Horn L.-C., Dorschner W. (2002). Smooth muscle cells from human urinary bladder express connexin 43 in vivo and in vitro. *World journal of urology*.

[26] Miyoshi H., Boyle M. B., MacKay L. B., Garfield R. E. (1996). Voltage-clamp studies of gap junctions between uterine muscle cells during term and preterm labor. *Biophysical Journal*.

[27] Leybaert L., Sanderson M. J. (2012). Intercellular Ca^2+^ waves: mechanisms and function. *Physiological Reviews*.

[28] Rouach N., Koulakoff A., Abudara V., Willecke K., Giaume C. (2008). Astroglial metabolic networks sustain hippocampal synaptic transmission. *Science*.

[29] Giaume C., Tabernero A., Medina J. M. (1997). Metabolic trafficking through astrocytic gap junctions. *Glia*.

[30] Maes M., Decrock E., Cogliati B. (2014). Connexin and pannexin (hemi)channels in the liver. *Frontiers in Physiology*.

[31] Batra N., Kar R., Jiang J. X. (2012). Gap junctions and hemichannels in signal transmission, function and development of bone. *Biochimica et Biophysica Acta: Biomembranes*.

[32] Decrock E., Krysko D. V., Vinken M. (2012). Transfer of IP_3_ through gap junctions is critical, but not sufficient, for the spread of apoptosis. *Cell Death and Differentiation*.

[33] Paul D. L., Ebihara L., Takemoto L. J., Swenson K. I., Goodenough D. A. (1991). Connexin46, a novel lens gap junction protein, induces voltage-gated currents in nonjunctional plasma membrane of Xenopus oocytes. *The Journal of Cell Biology*.

[34] Kang J., Kang N., Lovatt D. (2008). Connexin 43 hemichannels are permeable to ATP. *The Journal of Neuroscience*.

[35] Ye Z.-C., Wyeth M. S., Baltan-Tekkok S., Ransom B. R. (2003). Functional hemichannels in astrocytes: a novel mechanism of glutamate release. *Journal of Neuroscience*.

[36] Rana S., Dringen R. (2007). Gap junction hemichannel-mediated release of glutathione from cultured rat astrocytes. *Neuroscience Letters*.

[37] Goodenough D. A., Paul D. L. (2003). Beyond the gap: functions of unpaired connexon channels. *Nature Reviews Molecular Cell Biology*.

[38] Jiang J. X., Cherian P. P. (2003). Hemichannels formed by connexin 43 play an important role in the release of prostaglandin E(2) by osteocytes in response to mechanical strain. *Cell Communication and Adhesion*.

[39] Orellana J. A., Figueroa X. F., Sánchez H. A., Contreras-Duarte S., Velarde V., Sáez J. C. (2011). Hemichannels in the neurovascular unit and white matter under normal and inflamed conditions. *CNS and Neurological Disorders—Drug Targets*.

[40] D'hondt C., Iyyathurai J., Himpens B., Leybaert L., Bultynck G. (2014). Cx43-hemichannel function and regulation in physiology and pathophysiology: insights from the bovine corneal endothelial cell system and beyond. *Frontiers in Physiology*.

[41] Kamermans M., Fahrenfort I., Schultz K., Janssen-Bienhold U., Sjoerdsma T., Weiler R. (2001). Hemichannel-mediated inhibition in the outer retina. *Science*.

[42] Civitelli R. (2008). Cell-cell communication in the osteoblast/osteocyte lineage. *Archives of Biochemistry and Biophysics*.

[43] De Bock M., Culot M., Wang N. (2011). Connexin channels provide a target to manipulate brain endothelial calcium dynamics and blood-brain barrier permeability. *Journal of Cerebral Blood Flow and Metabolism*.

[44] Huckstepp R. T. R., IdBihi R., Eason R. (2010). Connexin hemichannel-mediated CO_2_-dependent release of ATP in the medulla oblongata contributes to central respiratory chemosensitivity. *The Journal of Physiology*.

[45] Wong C. W., Christen T., Roth I. (2006). Connexin37 protects against atherosclerosis by regulating monocyte adhesion. *Nature Medicine*.

[46] Karpuk N., Burkovetskaya M., Fritz T., Angle A., Kielian T. (2011). Neuroinflammation leads to region-dependent alterations in astrocyte gap junction communication and hemichannel activity. *Journal of Neuroscience*.

[47] O'Carroll S. J., Alkadhi M., Nicholson L. F. B., Green C. R. (2008). Connexin43 mimetic peptides reduce swelling, astrogliosis, and neuronal cell death after spinal cord injury. *Cell Communication and Adhesion*.

[48] Wang N., de Vuyst E., Ponsaerts R. (2013). Selective inhibition of Cx43 hemichannels by Gap19 and its impact on myocardial ischemia/reperfusion injury. *Basic Research in Cardiology*.

[49] Wang X., Ma A., Zhu W. (2013). The role of connexin 43 and hemichannels correlated with the astrocytic death following ischemia/reperfusion insult. *Cellular and Molecular Neurobiology*.

[50] Danesh-Meyer H. V., Kerr N. M., Zhang J. (2012). Connexin43 mimetic peptide reduces vascular leak and retinal ganglion cell death following retinal ischaemia. *Brain*.

[51] Danesh-Meyer H. V., Huang R., Nicholson L. F. B., Green C. R. (2008). Connexin43 antisense oligodeoxynucleotide treatment down-regulates the inflammatory response in an in vitro interphase organotypic culture model of optic nerve ischaemia. *Journal of Clinical Neuroscience*.

[52] Davidson J. O., Green C. R., B. Nicholson L. F. (2012). Connexin hemichannel blockade improves outcomes in a model of fetal ischemia. *Annals of Neurology*.

[53] Contreras J. E., Sánchez H. A., Véliz L. P., Bukauskas F. F., Bennett M. V. L., Sáez J. C. (2004). Role of connexin-based gap junction channels and hemichannels in ischemia-induced cell death in nervous tissue. *Brain Research Reviews*.

[54] Bargiotas P., Monyer H., Schwaninger M. (2009). Hemichannels in cerebral ischemia. *Current Molecular Medicine*.

[55] Decrock E., De Vuyst E., Vinken M. (2009). Connexin 43 hemichannels contribute to the propagation of apoptotic cell death in a rat C6 glioma cell model. *Cell Death and Differentiation*.

[56] Laird D. W. (2006). Life cycle of connexins in health and disease. *Biochemical Journal*.

[57] Choi D. H., Kim E.-M., Son H. J. (2008). A novel intracellular role of matrix metalloproteinase-3 during apoptosis of dopaminergic cells. *Journal of Neurochemistry*.

[58] Cauwe B., Martens E., Proost P., Opdenakker G. (2009). Multidimensional degradomics identifies systemic autoantigens and intracellular matrix proteins as novel gelatinase B/MMP-9 substrates. *Integrative Biology*.

[59] Hadler-Olsen E., Solli A. I., Hafstad A., Winberg J.-O., Uhlin-Hansen L. (2015). Intracellular MMP-2 activity in skeletal muscle is associated with type II fibers. *Journal of Cellular Physiology*.

[60] Soslau G., Mason C., Lynch S. (2013). Intracellular matrix metalloproteinase-2 (MMP-2) regulates human platelet activation via hydrolysis of talin. *Thrombosis and Haemostasis*.

[61] Sawicki G., Sanders E. J., Salas E., Wozniak M., Rodrigo J., Radomski M. W. (1998). Localization and translocation of MMP-2 during aggregation of human platelets. *Thrombosis and Haemostasis*.

[62] Vilen S.-T., Nyberg P., Hukkanen M. (2008). Intracellular co-localization of trypsin-2 and matrix metalloprotease-9: possible proteolytic cascade of trypsin-2, MMP-9 and enterokinase in carcinoma. *Experimental Cell Research*.

[63] Sawicki G. (2013). Intracellular regulation of matrix metalloproteinase-2 activity: new strategies in treatment and protection of heart subjected to oxidative stress. *Scientifica*.

[64] Frears E. R., Zhang Z., Blake D. R., O'Connell J. P., Winyard P. G. (1996). Inactivation of tissue inhibitor of metalloproteinase-1 by peroxynitrite. *FEBS Letters*.

[65] Lovett D. H., Mahimkar R., Raffai R. L. (2013). N-terminal truncated intracellular matrix metalloproteinase-2 induces cardiomyocyte hypertrophy, inflammation and systolic heart failure. *PLoS ONE*.

[66] Ali M. A. M., Chow A. K., Kandasamy A. D. (2012). Mechanisms of cytosolic targeting of matrix metalloproteinase-2. *Journal of Cellular Physiology*.

[67] Luo D., Mari B., Stoll I., Anglard P. (2002). Alternative splicing and promoter usage generates an intracellular stromelysin 3 isoform directly translated as an active matrix metalloproteinase. *The Journal of Biological Chemistry*.

[68] Fedarko N. S., Jain A., Karadag A., Fisher L. W. (2004). Three small integrin binding ligand N-linked glycoproteins (SIBLINGs) bind and activate specific matrix metalloproteinases. *The FASEB Journal*.

[69] Giovannone S., Remo B. F., Fishman G. I. (2012). Channeling diversity: gap junction expression in the heart. *Heart Rhythm*.

[70] Verheule S., Kaese S. (2013). Connexin diversity in the heart: insights from transgenic mouse models. *Frontiers in Pharmacology*.

[71] Liew R., Khairunnisa K., Gu Y. (2013). Role of tumor necrosis factor-alpha in the pathogenesis of atrial fibrosis and development of an arrhythmogenic substrate. *Circulation Journal*.

[72] Wang J., Li J.-S., Liu H.-Z. (2014). Dynamic alterations of connexin43, matrix metalloproteinase-2 and tissue inhibitor of matrix metalloproteinase-2 during ventricular fibrillation in canine. *Molecular and Cellular Biochemistry*.

[73] Givvimani S., Kundu S., Narayanan N. (2013). TIMP-2 mutant decreases MMP-2 activity and augments pressure overload induced LV dysfunction and heart failure. *Archives of Physiology and Biochemistry*.

[74] Peng H.-J., Dai D.-Z., Ji H., Dai Y. (2010). The separate roles of endothelin receptors participate in remodeling of matrix metalloproteinase and connexin 43 of cardiac fibroblasts in maladaptive response to isoproterenol. *European Journal of Pharmacology*.

[75] Tyagi N., Vacek J. C., Givvimani S., Sen U., Tyagi S. C. (2010). Cardiac specific deletion of *N*-methyl-d-aspartate receptor 1 ameliorates mtMMP-9 mediated autophagy/mitophagy in hyperhomocysteinemia. *Journal of Receptors and Signal Transduction*.

[76] Mohammad G., Kowluru R. A. (2011). Novel role of mitochondrial matrix metalloproteinase-2 in the development of diabetic retinopathy. *Investigative Ophthalmology and Visual Science*.

[77] Kundu S., Pushpakumar S. B., Tyagi A., Coley D., Sen U. (2013). Hydrogen sulfide deficiency and diabetic renal remodeling: role of matrix metalloproteinase-9. *The American Journal of Physiology—Endocrinology and Metabolism*.

[78] Wu X., Huang W., Luo G., Alain L. A. (2013). Hypoxia induces connexin 43 dysregulation by modulating matrix metalloproteinases via MAPK signaling. *Molecular and Cellular Biochemistry*.

[79] Song J., Tan H., Perry A. J. (2012). PROSPER: an integrated feature-based tool for predicting protease substrate cleavage sites. *PLoS ONE*.

[80] Verspurten J., Gevaert K., Declercq W., Vandenabeele P. (2009). SitePredicting the cleavage of proteinase substrates. *Trends in Biochemical Sciences*.

[81] Giepmans B. N. G., Verlaan I., Moolenaar W. H. (2001). Connexin-43 interactions with ZO-1 and alpha- and beta-tubulin. *Cell Communication and Adhesion*.

[82] Langlois S., Cowan K. N., Shao Q., Cowan B. J., Laird D. W. (2008). Caveolin-1 and -2 interact with connexin43 and regulate gap junctional intercellular communication in keratinocytes. *Molecular Biology of the Cell*.

[83] Saidi Brikci-Nigassa A., Clement M.-J., Ha-Duong T. (2012). Phosphorylation controls the interaction of the connexin43 C-terminal domain with tubulin and microtubules. *Biochemistry*.

[84] Retamal M. A., Cortés C. J., Reuss L., Bennett M. V. L., Sáez J. C. (2006). S-nitrosylation and permeation through connexin 43 hemichannels in astrocytes: induction by oxidant stress and reversal by reducing agents. *Proceedings of the National Academy of Sciences of the United States of America*.

[85] Johnstone S. R., Billaud M., Lohman A. W., Taddeo E. P., Isakson B. E. (2012). Posttranslational modifications in connexins and pannexins. *Journal of Membrane Biology*.

[86] Yahuaca P., Ek-Vitorin J. F., Rush P., Delmar M., Taffet S. M. (2000). Identification of a protein kinase activity that phosphorylates connexin43 in a pH-dependent manner. *Brazilian Journal of Medical and Biological Research*.

[87] Duffy H. S., Sorgen P. L., Girvin M. E. (2002). pH-dependent intramolecular binding and structure involving Cx43 cytoplasmic domains. *The Journal of Biological Chemistry*.

[88] Seki A., Duffy H. S., Coombs W., Spray D. C., Taffet S. M., Delmar M. (2004). Modifications in the biophysical properties of connexin43 channels by a peptide of the cytoplasmic loop region. *Circulation Research*.

[89] Delmar M., Coombs W., Sorgen P., Duffy H. S., Taffet S. M. (2004). Structural bases for the chemical regulation of Connexin43 channels. *Cardiovascular Research*.

[90] Ek-Vitorín J. F., Calero G., Morley G. E., Coombs W., Taffet S. M., Delmar M. (1996). pH regulation of connexin43: molecular analysis of the gating particle. *Biophysical Journal*.

[91] Bukauskas F. F., Jordan K., Bukauskiene A. (2000). Clustering of connexin 43-enhanced green fluorescent protein gap junction channels and functional coupling in living cells. *Proceedings of the National Academy of Sciences of the United States of America*.

[92] Anumonwo J. M. B., Taffet S. M., Gu H., Chanson M., Moreno A. P., Delmar M. (2001). The carboxyl terminal domain regulates the unitary conductance and voltage dependence of connexin40 gap junction channels. *Circulation Research*.

[93] Moreno A. P., Chanson M., Anumonwo J. (2002). Role of the carboxyl terminal of connexin43 in transjunctional fast voltage gating. *Circulation Research*.

[94] Shibayama J., Gutiérrez C., González D. (2006). Effect of charge substitutions at residue His-142 on voltage gating of connexin43 channels. *Biophysical Journal*.

[95] Ek J. F., Delmar M., Perzova R., Taffet S. M. (1994). Role of histidine 95 on pH gating of the cardiac gap junction protein connexin43. *Circulation Research*.

[96] Wang N., De Bock M., Decrock E. (2013). Connexin targeting peptides as inhibitors of voltage- and intracellular Ca^2+^-triggered Cx43 hemichannel opening. *Neuropharmacology*.

[97] Wang N., De Bock M., Antoons G. (2012). Connexin mimetic peptides inhibit Cx43 hemichannel opening triggered by voltage and intracellular Ca^2+^ elevation. *Basic Research in Cardiology*.

[98] De Vuyst E., Wang N., Decrock E. (2009). Ca^2+^ regulation of connexin 43 hemichannels in C6 glioma and glial cells. *Cell Calcium*.

[99] Shintani-Ishida K., Uemura K., Yoshida K.-I. (2007). Hemichannels in cardiomyocytes open transiently during ischemia and contribute to reperfusion injury following brief ischemia. *American Journal of Physiology—Heart and Circulatory Physiology*.

[100] Ponsaerts R., De Vuyst E., Retamal M. (2010). Intramolecular loop/tail interactions are essential for connexin 43-hemichannel activity. *The FASEB Journal*.

[101] Ponsaerts R., D'hondt C., Hertens F. (2012). RhoA GTPase switch controls Cx43-hemichannel activity through the contractile system. *PLoS ONE*.

[102] Iyyathurai J., D'Hondt C., Wang N. (2013). Peptides and peptide-derived molecules targeting the intracellular domains of Cx43: gap junctions versus hemichannels. *Neuropharmacology*.

[103] Falk M. M. (2000). Cell-free synthesis for analyzing the membrane integration, oligomerization, and assembly characteristics of gap junction connexins. *Methods*.

[104] VanSlyke J. K., Naus C. C., Musil L. S. (2009). Conformational maturation and post-er multisubunit assembly of gap junction proteins. *Molecular Biology of the Cell*.

[105] Gaietta G., Deerinck T. J., Adams S. R. (2002). Multicolor and electron microscopic imaging of connexin trafficking. *Science*.

[106] Lampe P. D., Lau A. F. (2004). The effects of connexin phosphorylation on gap junctional communication. *The International Journal of Biochemistry & Cell Biology*.

[107] Maass K., Shibayama J., Chase S. E., Willecke K., Delmar M. (2007). C-terminal truncation of connexin43 changes number, size, and localization of cardiac gap junction plaques. *Circulation Research*.

[108] Unger V. M., Kumar N. M., Gilula N. B., Yeager M. (1999). Three-dimensional structure of a recombinant gap junction membrane channel. *Science*.

[109] Kang T.-C., Kim D.-S., Kwak S.-E. (2006). Epileptogenic roles of astroglial death and regeneration in the dentate gyrus of experimental temporal lobe epilepsy. *Glia*.

[110] Gong X.-Q., Shao Q., Langlois S., Bai D., Laird D. W. (2007). Differential potency of dominant negative connexin43 mutants in oculodentodigital dysplasia. *The Journal of Biological Chemistry*.

[111] Lai A., Le D.-N., Paznekas W. A., Gifford W. D., Jabs E. W., Charles A. C. (2006). Oculodentodigital dysplasia connexin43 mutations result in non-functional connexin hemichannels and gap junctions in C6 glioma cells. *Journal of Cell Science*.

[112] Churko J. M., Shao Q., Gong X.-Q. (2011). Human dermal fibroblasts derived from oculodentodigital dysplasia patients suggest that patients may have wound-healing defects. *Human Mutation*.

[113] Hong H. M., Yang J. J., Shieh J. C., Lin M. L., Li S. Y. (2010). Novel mutations in the connexin43 (GJA1) and GJA1 pseudogene may contribute to nonsyndromic hearing loss. *Human Genetics*.

[114] De Vuyst E., Decrock E., De Bock M. (2007). Connexin hemichannels and gap junction channels are differentially influenced by lipopolysaccharide and basic fibroblast growth factor. *Molecular Biology of the Cell*.

[115] Lübkemeier I., Requardt R. P., Lin X. (2013). Deletion of the last five C-terminal amino acid residues of connexin43 leads to lethal ventricular arrhythmias in mice without affecting coupling via gap junction channels. *Basic Research in Cardiology*.

[116] Kozoriz M. G., Bechberger J. F., Bechberger G. R. (2010). The connexin43 C-terminal region mediates neuroprotection during stroke. *Journal of Neuropathology and Experimental Neurology*.

[117] Morley G. E., Taffet S. M., Delmar M. (1996). Intramolecular interactions mediate pH regulation of connexin43 channels. *Biophysical Journal*.

[118] Hunter A. W., Barker R. J., Zhu C., Gourdie R. G. (2005). Zonula occludens-1 alters connexin43 gap junction size and organization by influencing channel accretion. *Molecular Biology of the Cell*.

[119] Rhett J. M., Jourdan J., Gourdie R. G. (2011). Connexin 43 connexon to gap junction transition is regulated by zonula occludens-1. *Molecular Biology of the Cell*.

[120] Soder B. L., Propst J. T., Brooks T. M. (2009). The connexin43 carboxyl-terminal peptide ACT1 modulates the biological response to silicone implants. *Plastic and Reconstructive Surgery*.

[121] Ghatnekar G. S., Grek C. L., Armstrong D. G., Desai S. C., Gourdie R. G. (2015). The effect of a connexin43-based Peptide on the healing of chronic venous leg ulcers: a multicenter, randomized trial. *Journal of Investigative Dermatology*.

[122] Grek C. L., Prasad G. M., Viswanathan V., Armstrong D. G., Gourdie R. G., Ghatnekar G. S. (2015). Topical administration of a connexin43-based peptide augments healing of chronic neuropathic diabetic foot ulcers: a multicenter, randomized trial. *Wound Repair and Regeneration*.

[123] Moore K., Ghatnekar G., Gourdie R. G., Potts J. D. (2014). Impact of the controlled release of a connexin 43 peptide on corneal wound closure in an STZ model of type I diabetes. *PLoS ONE*.

[124] O'Quinn M. P., Palatinus J. A., Harris B. S., Hewett K. W., Gourdie R. G. (2011). A peptide mimetic of the connexin43 carboxyl terminus reduces gap junction remodeling and induced arrhythmia following ventricular injury. *Circulation Research*.

[125] Grek C. L., Rhett J. M., Bruce J. S., Abt M. A., Ghatnekar G. S., Yeh E. S. (2015). Targeting connexin 43 with *α*–connexin carboxyl-terminal (ACT1) peptide enhances the activity of the targeted inhibitors, tamoxifen and lapatinib, in breast cancer: clinical implication for ACT1. *BMC Cancer*.

[126] Santiago M. F., Alcami P., Striedinger K. M., Spray D. C., Scemes E. (2010). The carboxyl-terminal domain of connexin43 is a negative modulator of neuronal differentiation. *Journal of Biological Chemistry*.

[127] Dang X., Doble B. W., Kardami E. (2003). The carboxy-tail of connexin-43 localizes to the nucleus and inhibits cell growth. *Molecular and Cellular Biochemistry*.

[128] Moorby C., Patel M. (2001). Dual functions for connexins: Cx43 regulates growth independently of gap junction formation. *Experimental Cell Research*.

[129] Vinken M., Decrock E., Leybaert L. (2012). Non-channel functions of connexins in cell growth and cell death. *Biochimica et Biophysica Acta—Biomembranes*.

[130] Johansen D., Cruciani V., Sundset R., Ytrehus K., Mikalsen S.-O. (2011). Ischemia induces closure of gap junctional channels and opening of hemichannels in heart-derived cells and tissue. *Cellular Physiology and Biochemistry*.

[131] Joshi-Mukherjee R., Coombs W., Burrer C., Alvarez de Mora I., Delmar M., Taffet S. M. (2007). Evidence for the presence of a free C-Terminal fragment of Cx43 in cultured cells. *Cell Communication and Adhesion*.

[132] Smyth J. W., Shaw R. M. (2013). Autoregulation of connexin43 gap junction formation by internally translated isoforms. *Cell Reports*.

[133] Salat-Canela C., Sesé M., Peula C., Ramón Y Cajal S., Aasen T. (2014). Internal translation of the connexin 43 transcript. *Cell Communication and Signaling*.

[134] Salat-Canela C., Muñoz M., Sesé M., Ramón y Cajal S., Aasen T. (2015). Post-transcriptional regulation of connexins. *Biochemical Society Transactions*.

[135] Schulz R., Görge P. M., Görbe A., Ferdinandy P., Lampe P. D., Leybaert L. (2015). Connexin 43 is an emerging therapeutic target in ischemia/reperfusion injury, cardioprotection and neuroprotection. *Pharmacology & Therapeutics*.

[136] Hawat G., Hélie P., Baroudi G. (2012). Single intravenous low-dose injections of connexin 43 mimetic peptides protect ischemic heart in vivo against myocardial infarction. *Journal of Molecular and Cellular Cardiology*.

[137] Yoon J. J., Green C. R., O'Carroll S. J., Nicholson L. F. B. (2010). Dose-dependent protective effect of connexin43 mimetic peptide against neurodegeneration in an ex vivo model of epileptiform lesion. *Epilepsy Research*.

[138] Chen Y.-S., Toth I., Danesh-Meyer H. V., Green C. R., Rupenthal I. D. (2013). Cytotoxicity and vitreous stability of chemically modified connexin43 mimetic peptides for the treatment of optic neuropathy. *Journal of Pharmaceutical Sciences*.

[139] Orellana J. A., Hernández D. E., Ezan P. (2010). Hypoxia in high glucose followed by reoxygenation in normal glucose reduces the viability of cortical astrocytes through increased permeability of connexin 43 hemichannels. *Glia*.

[140] Umebayashi D., Natsume A., Takeuchi H. (2014). Blockade of gap junction hemichannel protects secondary spinal cord injury from activated microglia-mediated glutamate exitoneurotoxicity. *Journal of Neurotrauma*.

[141] Orellana J. A., Shoji K. F., Abudara V. (2011). Amyloid *β*-induced death in neurons involves glial and neuronal hemichannels. *Journal of Neuroscience*.

[142] Shijie J., Takeuchi H., Yawata I. (2009). Blockade of glutamate release from microglia attenuates experimental autoimmune encephalomyelitis in mice. *Tohoku Journal of Experimental Medicine*.

[143] Huang C., Han X., Li X. (2012). Critical role of connexin 43 in secondary expansion of traumatic spinal cord injury. *Journal of Neuroscience*.

[144] Bennett M. V. L., Garré J. M., Orellana J. A., Bukauskas F. F., Nedergaard M., Sáez J. C. (2012). Connexin and pannexin hemichannels in inflammatory responses of glia and neurons. *Brain Research*.

[145] Shaikh S. B., Uy B., Perera A., Nicholson L. F. B. (2012). AGEs-RAGE mediated up-regulation of connexin43 in activated human microglial CHME-5 cells. *Neurochemistry International*.

[146] Retamal M. A., Froger N., Palacios-Prado N. (2007). Cx43 hemichannels and gap junction channels in astrocytes are regulated oppositely by proinflammatory cytokines released from activated microglia. *The Journal of Neuroscience*.

[147] Morita M., Saruta C., Kozuka N. (2007). Dual regulation of astrocyte gap junction hemichannels by growth factors and a pro-inflammatory cytokine via the mitogen-activated protein kinase cascade. *Glia*.

[148] Xiong J., Burkovetskaya M., Karpuk N., Kielian T. (2012). IL-1RI (interleukin-1 receptor type I) signalling is essential for host defence and hemichannel activity during acute central nervous system bacterial infection. *ASN Neuro*.

[149] Wang N., De Bock M., Decrock E. (2013). Paracrine signaling through plasma membrane hemichannels. *Biochimica et Biophysica Acta*.

[150] Arcuino G., Lin J. H.-C., Takano T. (2002). Intercellular calcium signaling mediated by point-source burst release of ATP. *Proceedings of the National Academy of Sciences of the United States of America*.

[151] Lecut C., Frederix K., Johnson D. M. (2009). P2X1 ion channels promote neutrophil chemotaxis through Rho kinase activation. *Journal of Immunology*.

[152] Sumi Y., Woehrle T., Chen Y. (2014). Plasma ATP is required for neutrophil activation in a mouse sepsis model. *Shock*.

[153] Riteau N., Gasse P., Fauconnier L. (2010). Extracellular ATP is a danger signal activating P2X7 receptor in lung inflammation and fibrosis. *American Journal of Respiratory and Critical Care Medicine*.

[154] Chen Y., Corriden R., Inoue Y. (2006). ATP release guides neutrophil chemotaxis via P2Y2 and A3 receptors. *Science*.

[155] Calder B. W., Rhett J. M., Bainbridge H., Fann S. A., Gourdie R. G., Yost M. J. (2015). Inhibition of connexin 43 hemichannel-mediated ATP release attenuates early inflammation during the foreign body response. *Tissue Engineering Part A*.

[156] Eltzschig H. K., Eckle T., Mager A. (2006). ATP release from activated neutrophils occurs via connexin 43 and modulates adenosine-dependent endothelial cell function. *Circulation Research*.

[157] Eltzschig H. K., MacManus C. F., Colgan S. P. (2008). Neutrophils as sources of extracellular nucleotides: functional consequences at the vascular interface. *Trends in Cardiovascular Medicine*.

[158] Gombault A., Baron L., Couillin I. (2012). ATP release and purinergic signaling in NLRP3 inflammasome activation. *Frontiers in Immunology*.

[159] Robertson J., Lang S., Lambert P. A., Martin P. E. (2010). Peptidoglycan derived from *Staphylococcus epidermidis* induces Connexin43 hemichannel activity with consequences on the innate immune response in endothelial cells. *Biochemical Journal*.

[160] Chen G., Park C.-K., Xie R.-G., Berta T., Nedergaard M., Ji R.-R. (2014). Connexin-43 induces chemokine release from spinal cord astrocytes to maintain late-phase neuropathic pain in mice. *Brain*.

[161] Lee J. Y., Choi H. Y., Yune T. Y. (2015). MMP-3 secreted from endothelial cells of blood vessels after spinal cord injury activates microglia, leading to oligodendrocyte cell death. *Neurobiology of Disease*.

[162] Lee J. Y., Choi H. Y., Na W. H., Ju B. G., Yune T. Y. (2015). 17*β*-estradiol inhibits MMP-9 and SUR1/TrpM4 expression and activation and thereby attenuates BSCB disruption/hemorrhage after spinal cord injury in male rats. *Endocrinology*.

[163] Zinnhardt B., Viel T., Wachsmuth L. (2015). Multimodal imaging reveals temporal and spatial microglia and matrix metalloproteinase activity after experimental stroke. *Journal of Cerebral Blood Flow & Metabolism*.

[164] Pollock E., Everest M., Brown A., Poulter M. O. (2014). Metalloproteinase inhibition prevents inhibitory synapse reorganization and seizure genesis. *Neurobiology of Disease*.

[165] Mizoguchi H., Yamada K. (2013). Roles of matrix metalloproteinases and their targets in epileptogenesis and seizures. *Clinical Psychopharmacology and Neuroscience*.

[166] Zhang X., Cheng M., Chintala S. K. (2004). Optic nerve ligation leads to astrocyte-associated matrix metalloproteinase-9 induction in the mouse retina. *Neuroscience Letters*.

[167] Corry D. B., Kiss A., Song L.-Z. (2004). Overlapping and independent contributions of MMP2 and MMP9 to lung allergic inflammatory cell egression through decreased CC chemokines. *The FASEB Journal*.

[168] Corry D. B., Rishi K., Kanellis J. (2002). Decreased allergic lung inflammatory cell egression and increased susceptibility to asphyxiation in MMP2-deficiency. *Nature Immunology*.

[169] Deatrick K. B., Luke C. E., Elfline M. A. (2013). The effect of matrix metalloproteinase 2 and matrix metalloproteinase 2/9 deletion in experimental post-thrombotic vein wall remodeling. *Journal of Vascular Surgery*.

[170] Cataldo D. D., Tournoy K. G., Vermaelen K. (2002). Matrix metalloproteinase-9 deficiency impairs cellular infiltration and bronchial hyperresponsiveness during allergen-induced airway inflammation. *The American Journal of Pathology*.

[171] Luttun A., Lutgens E., Manderveld A. (2004). Loss of matrix metalloproteinase-9 or matrix metalloproteinase-12 protects apolipoprotein E-deficient mice against atherosclerotic media destruction but differentially affects plaque growth. *Circulation*.

[172] Li Q., Park P. W., Wilson C. L., Parks W. C. (2002). Matrilysin shedding of syndecan-1 regulates chemokine mobilization and transepithelial efflux of neutrophils in acute lung injury. *Cell*.

[173] Mitsiades N., Yu W.-H., Poulaki V., Tsokos M., Stamenkovic I. (2001). Matrix metalloproteinase-7-mediated cleavage of Fas ligand protects tumor cells from chemotherapeutic drug cytotoxicity. *Cancer Research*.

[174] Tian W., Kyriakides T. R. (2009). Matrix metalloproteinase-9 deficiency leads to prolonged foreign body response in the brain associated with increased IL-1beta levels and leakage of the blood-brain barrier. *Matrix Biology*.

[175] McMillan S. J., Kearley J., Campbell J. D. (2004). Matrix metalloproteinase-9 deficiency results in enhanced allergen-induced airway inflammation. *Journal of Immunology*.

[176] Lanone S., Zheng T., Zhu Z. (2002). Overlapping and enzyme-specific contributions of matrix metalloproteinases-9 and -12 in IL-13-induced inflammation and remodeling. *The Journal of Clinical Investigation*.

[177] Sáez P. J., Shoji K. F., Retamal M. A. (2013). ATP is required and advances cytokine-induced gap junction formation in microglia in vitro. *Mediators of Inflammation*.

[178] Froger N., Orellana J. A., Calvo C.-F. (2010). Inhibition of cytokine-induced connexin43 hemichannel activity in astrocytes is neuroprotective. *Molecular and Cellular Neuroscience*.

[179] Wu C.-Y., Hsieh H.-L., Sun C.-C., Yang C.-M. (2009). IL-1*β* induces MMP-9 expression via a Ca^2+^-dependent CaMKII/JNK/c-Jun cascade in rat brain astrocytes. *Glia*.

[180] Hirst-Jensen B. J., Sahoo P., Kieken F., Delmar M., Sorgen P. L. (2007). Characterization of the pH-dependent interaction between the gap junction protein connexin43 carboxyl terminus and cytoplasmic loop domains. *Journal of Biological Chemistry*.

[181] Liu F., Arce F. T., Ramachandran S., Lal R. (2006). Nanomechanics of hemichannel conformations: connexin flexibility underlying channel opening and closing. *Journal of Biological Chemistry*.

[182] Butkevich E., Hülsmann S., Wenzel D., Shirao T., Duden R., Majoul I. (2004). Drebrin is a novel connexin-43 binding partner that links gap junctions to the submembrane cytoskeleton. *Current Biology*.

[183] Vitale M. L., Akpovi C. D., Pelletier R.-M. (2009). Cortactin/tyrosine-phosphorylated cortactin interaction with connexin 43 in mouse seminiferous tubules. *Microscopy Research and Technique*.

[184] Pidoux G., Gerbaud P., Dompierre J. (2014). A PKA-ezrin-Cx43 signaling complex controls gap junction communication and thereby trophoblast cell fusion. *Journal of Cell Science*.

[185] Sorgen P. L., Duffy H. S., Sahoo P., Coombs W., Delmar M., Spray D. C. (2004). Structural changes in the carboxyl terminus of the gap junction protein connexin43 indicates signaling between binding domains for c-Src and zonula occludens-1. *The Journal of Biological Chemistry*.

[186] Lan Z., Kurata W. E., Martyn K. D., Jin C., Lau A. F. (2005). Novel Rab GAP-like protein, CIP85, interacts with connexin43 and induces its degradation. *Biochemistry*.

[187] Wun C. S., Bechberger J. F., Rushlow W. J., Naus C. C. (2008). Dose-dependent differential upregulation of CCN1/Cyr61 and CCN3/NOV by the gap junction protein connexin43 in glioma cells. *Journal of Cellular Biochemistry*.

[188] Giepmans B. N. G., Moolenaar W. H. (1998). The gap junction protein connexin43 interacts with the second PDZ domain of the zona occludens-1 protein. *Current Biology*.

[189] Ponsaerts R., Wang N., Himpens B., Leybaert L., Bultynck G. (2012). The contractile system as a negative regulator of the connexin 43 hemichannel. *Biology of the Cell*.

[190] Ermak G., Davies K. J. A. (2002). Calcium and oxidative stress: from cell signaling to cell death. *Molecular Immunology*.

[191] Ramachandra S., Xie L.-H., John S. A., Subramaniam S., Lal R. (2007). A novel role for connexin hemichannel in oxidative stress and smoking-induced cell injury. *PLoS ONE*.

[192] Kar R., Riquelme M. A., Werner S., Jiang J. X. (2013). Connexin 43 channels protect osteocytes against oxidative stress-induced cell death. *Journal of Bone and Mineral Research*.

[193] Cherian P. P., Siller-Jackson A. J., Gu S. (2005). Mechanical strain opens connexin 43 hemichannels in osteocytes: a novel mechanism for the release of prostaglandin. *Molecular Biology of the Cell*.

[194] Plotkin L. I., Manolagas S. C., Bellido T. (2002). Transduction of cell survival signals by connexin-43 hemichannels. *The Journal of Biological Chemistry*.

[195] Lee D. E., Shin B. J., Hur H. J. (2010). Quercetin, the active phenolic component in kiwifruit, prevents hydrogen peroxide-induced inhibition of gap-junction intercellular communication. *British Journal of Nutrition*.

[196] Sovari A. A., Rutledge C. A., Jeong E.-M. (2013). Mitochondria oxidative stress, connexin43 remodeling, and sudden arrhythmic death. *Circulation: Arrhythmia and Electrophysiology*.

[197] John G. R., Scemes E., Suadicani S. O. (1999). IL-1*β* differentially regulates calcium wave propagation between primary human fetal astrocytes via pathways involving P2 receptors and gap junction channels. *Proceedings of the National Academy of Sciences of the United States of America*.

[198] Duffy H. S., John G. R., Lee S. C., Brosnan C. F., Spray D. C. (2000). Reciprocal regulation of the junctional proteins claudin-1 and connexin43 by interleukin-1beta in primary human fetal astrocytes. *The Journal of Neuroscience*.

[199] Même W., Ezan P., Venance L., Glowinski J., Giaume C. (2004). ATP-induced inhibition of gap junctional communication is enhanced by interleukin-1 beta treatment in cultured astrocytes. *Neuroscience*.

[200] Castellano P., Eugenin E. A. (2014). Regulation of gap junction channels by infectious agents and inflammation in the CNS. *Frontiers in Cellular Neuroscience*.

[201] Kasper C. A., Sorg I., Schmutz C. (2010). Cell-cell propagation of NF-kappaB transcription factor and MAP kinase activation amplifies innate immunity against bacterial infection. *Immunity*.

[202] Eugenín E. A., Brañes M. C., Berman J. W., Sáez J. C. (2003). TNF-*αα* plus IFN-*γ* induce connexin43 expression and formation of gap junctions between human monocytes/macrophages that enhance physiological responses. *Journal of Immunology*.

[203] Feine I., Pinkas I., Salomon Y., Scherz A. (2012). Local oxidative stress expansion through endothelial cells—a key role for gap junction intercellular communication. *PLoS ONE*.

[204] Neijssen J., Herberts C., Drijfhout J. W., Reits E., Janssen L., Neefjes J. (2005). Cross-presentation by intercellular peptide transfer through gap junctions. *Nature*.

[205] Hervé J.-C., Bourmeyster N., Sarrouilhe D., Duffy H. S. (2007). Gap junctional complexes: from partners to functions. *Progress in Biophysics and Molecular Biology*.

[206] Tabernero A., Gangoso E., Jaraíz-Rodríguez M., Medina J. (2015). The role of connexin43–Src interaction in astrocytomas: a molecular puzzle. *Neuroscience*.

[207] Scemes E., Duval N., Meda P. (2003). Reduced expression of P2Y_1_ receptors in connexin43-null mice alters calcium signaling and migration of neural progenitor cells. *Journal of Neuroscience*.

[208] Scemes E. (2008). Modulation of astrocyte P2Y1 receptors by the carboxyl terminal domain of the gap junction protein Cx43. *Glia*.

[209] Li M. W. M., Mruk D. D., Cheng C. Y. (2013). Gap junctions and blood-tissue barriers. *Advances in Experimental Medicine and Biology*.

[210] Li M. W. M., Mruk D. D., Lee W. M., Cheng C. Y. (2010). Connexin 43 is critical to maintain the homeostasis of the blood-testis barrier via its effects on tight junction reassembly. *Proceedings of the National Academy of Sciences of the United States of America*.

[211] Morita H., Katsuno T., Hoshimoto A., Hirano N., Saito Y., Suzuki Y. (2004). Connexin 26-mediated gap junctional intercellular communication suppresses paracellular permeability of human intestinal epithelial cell monolayers. *Experimental Cell Research*.

[212] Kojima T., Murata M., Go M., Spray D. C., Sawada N. (2007). Connexins induce and maintain tight junctions in epithelial cells. *Journal of Membrane Biology*.

[213] Wu J.-C., Tsai R.-Y., Chung T.-H. (2003). Role of catenins in the development of gap junctions in rat cardiomyocytes. *Journal of Cellular Biochemistry*.

[214] Wei C.-J., Francis R., Xu X., Lo C. W. (2005). Connexin43 associated with an N-cadherin-containing multiprotein complex is required for gap junction formation in NIH_3_T_3_ cells. *The Journal of Biological Chemistry*.

